# The influence of physical activity on internet addiction among Chinese college students: the mediating role of self-esteem and the moderating role of gender

**DOI:** 10.1186/s12889-024-18474-1

**Published:** 2024-04-01

**Authors:** Du Zhihao, Wang Tao, Sun Yingjie, Zhai Feng

**Affiliations:** 1 Jishou University, 416000 Jishou, Hunan China; 2 College of Physical Education, China University of Mining and Technology, 221116 Xuzhou, Jiangsu China; 3 College of Physical Education, Zhengzhou University, 451000 Zhengzhou, Henan, China

**Keywords:** Physical activity, Self-esteem, Internet addiction, Mediating effect, Moderating effect

## Abstract

**Objectives:**

The significance of self-esteem in the relationship between physical activity and Internet addiction among college students cannot be over, as it lays a solid foundation for the prevention and control of Internet addiction.

**Methods:**

A total of 950 college students were surveyed using the Physical Activity Rating Scale (PARS-3), Rosenberg Self-Esteem Scale (SES), and Chinese Internet Addiction Scale (CIAS-R) through a cluster random sampling method. Descriptive statistics, independent sample t-test, partial correlation analysis, mediation effect, moderation effect, and Bootstrap testing were conducted on the collected data to analyze and interpret the results.

**Results:**

(1) Significant gender differences were found in the amount of physical activity and the degree of Internet addiction among college students(P&& lt;0.001); (2) Physical activity was significantly and positively correlated with self-esteem (*r* = 0.26, *P* < 0.001), but significantly and negatively correlated with Internet addiction (*r*=-0.23, *P* < 0.001); Meanwhile, self-esteem and Internet addiction were significantly and negatively related to self-esteem (*r*=-0.22, *P* < 0.001). (3) Mediating effect analyses showed that self-esteem played a partial mediating role in physical activity and Internet addiction among college students, accounting for a portion of 78.95%. (4) A moderating effect of gender on the relationship between physical activity and Internet addiction was discovered.

**Conclusion:**

The physical activity level of male students is significantly higher than that of female students, while the degree of internet addiction among female students is notably higher than that of male students. Physical activity can not only directly improve the issue of internet addiction among college students, but also indirectly improve it through self-esteem, with gender playing a moderating role in this process. This conclusion has practical reference significance for preventing and controlling internet addiction among college students, and provides evidence support for using physical activity as a reference solution in clinical applications. Additionally, it suggests that gender should be taken into account when preventing and intervening in internet addiction among college students, and different strategies and methods should be adopted for male and female students. Male students should be encouraged to participate more in physical activities, gradually increasing the frequency, duration, and intensity of their participation, in order to divert their attention and enhance their sense of achievement in sports, thereby reducing their use of mobile phones. For female students, on the other hand, it is important to strengthen real-life communication, change the form of sports participation, engage in group, collaborative, and different situational sports activities, and enhance their focus and attention in sports, in order to reduce their internet dependency, better guide them to use the internet reasonably, and enable them to achieve emotional release through sports.

## Background

By 2020, China’s total population has reached 940 million and the Internet penetration rate reached 67.0%, which is at an extremely high level [1]. The Internet penetration rate is strongly linked to symptoms of Internet addiction among college students. This is because the Internet can fulfill college students’ needs in their daily study, life, and social interactions. This fulfillment can inevitably lead to psychological independence and Internet addiction. According to Yiman Liu’s (2021) meta-study analysis, the detection rate of Internet addiction among college students during 2011-2018Ranged from 10.2–13.2% [2], and the detection rate of Internet addiction during the epidemic was 32.4% [3]. This clearly suggests that Internet addiction has become a hard nut to crack among Chinese college students. Internet addiction is often accompanied by various psychological and behavioral problems, such as emotional disorders [4, 5], physical health [6], sleep disorders [7], academic failure [8], and suicidal behavior [9]. Consequently, to prevent and cope with Internet addiction among college students demands immediate action.

Numerous studies have confirmed a significant negative correlation between physical activity and Internet addiction [10]. This means that the more physically active people are, the lower their Internet addiction symptoms tend to be. Meanwhile, physical activity can be utilized as a treatment for Internet addiction [10]. A multitude of prior intervention studies have confirmed that physical activity can significantly improve Internet addiction symptoms [11]. For example, two Meta-analyses by Jim Wu (2018, 2019) which include 13 and 22 RCTs and erects with sample sizes of 720 and 2,838 respectively, both conclude that interventions of physical activity have a significant effect on adolescents’ internet addiction, which also supports the above viewpoints. In addition, by comparing the effectiveness of exercise and other measures, the final results of their reticulated Meta-analyses showed that physical exercise has the highest likelihood of being the optimal measure to intervene in adolescents’ Internet addiction [12, 13]. Accordingly, this study proposes the hypothesis that physical activity is one of the protective resolutions for Internet addiction.

Self-esteem is an individual’s holistic sense of self-value as well as a comprehensive evaluation of personal value [14]. The role of physical activity in building up individual’s self-esteem has been demonstrated on several occasions. Spence et al. (2005) conducted a Meta-analysis of more than 100 studies exploring the relationship between physical activity and self-esteem in adults, and results showed that physical activity increased overall self-esteem and physical self-esteem of the adults [15].Ozsaker Dorak et al. (2012) used an experimental intervention to demonstrate that children who were actively involved in physical activity had higher self-esteem [16]. Chinese scholar Sun Chao’s (2020) study found that physical activity can promote the level of self-esteem [17].The research on the relationship between self-esteem and Internet addiction, Bahrainian et al. (2014) concluded that some depression-related personalities, such as low self-esteem, are important contributors to Internet addiction [18]. Self-esteem can not only directly affect Internet addiction, but also indirectly affecting other variables [19]. Additionally, numerous studies have confirmed that self-esteem is negatively correlated with Internet addiction [20, 21]. This means that people with low self-esteem usually have a higher tendency of Internet addiction. In addition, current research has not found any study directly exploring the mediating role of self-esteem between physical activity and Internet addiction. Instead, most studies investigate the role of self-esteem in Internet addiction from other psychological perspectives, such as the family atmosphere [22], the need to belong [23], and the executive function [24]. However, all of the above studies may imply, either positively or negatively, that physical activity can increase individual’s level of self-esteem, and that the level of self-esteem is negatively related to Internet addiction. As a result, this study proposes the hypothesis that self-esteem mediates the effect between physical activity and Internet addiction.

Gender differences in Internet addiction have been validated many times. The study by Zhang Jinjian et al. (2023) further validated gender differences in Internet addiction by examining the dynamic developmental trajectory of Internet addiction among college students, and a gender effect in Internet addiction among college students was found [25]. For instance, a previous study found that men are more likely to be addicted to the Internet than women [26]. In terms of the degree of Internet addiction, Italian scholar Antonio surveyed 1,035 students from three cities in southern Italy, and the study showed that the index of Internet addiction of men was significantly higher than that of women [27]. However, some scholars also found that women’s Internet addiction is significantly greater than men’s [28], so there is still a controversy over whether men or women are more addicted to Internet. Therefore, this paper will further validate the gender differences between men and women in Internet addiction. At the same time, all the above views indicate that gender should be considered in the study of Internet addiction, and is often used as a moderating variable. According to the psychological theory of gender, men and women also have certain differences in the amount of physical activity, the type of sports programs they participate in, and their sports performance [29]. Researchers have found that there are significant differences in participation of physical activity between genders [30]. Based on this, this study proposed the hypothesis that gender plays a moderating role between physical activity and internet addiction among college students.

**Fig. 1:**
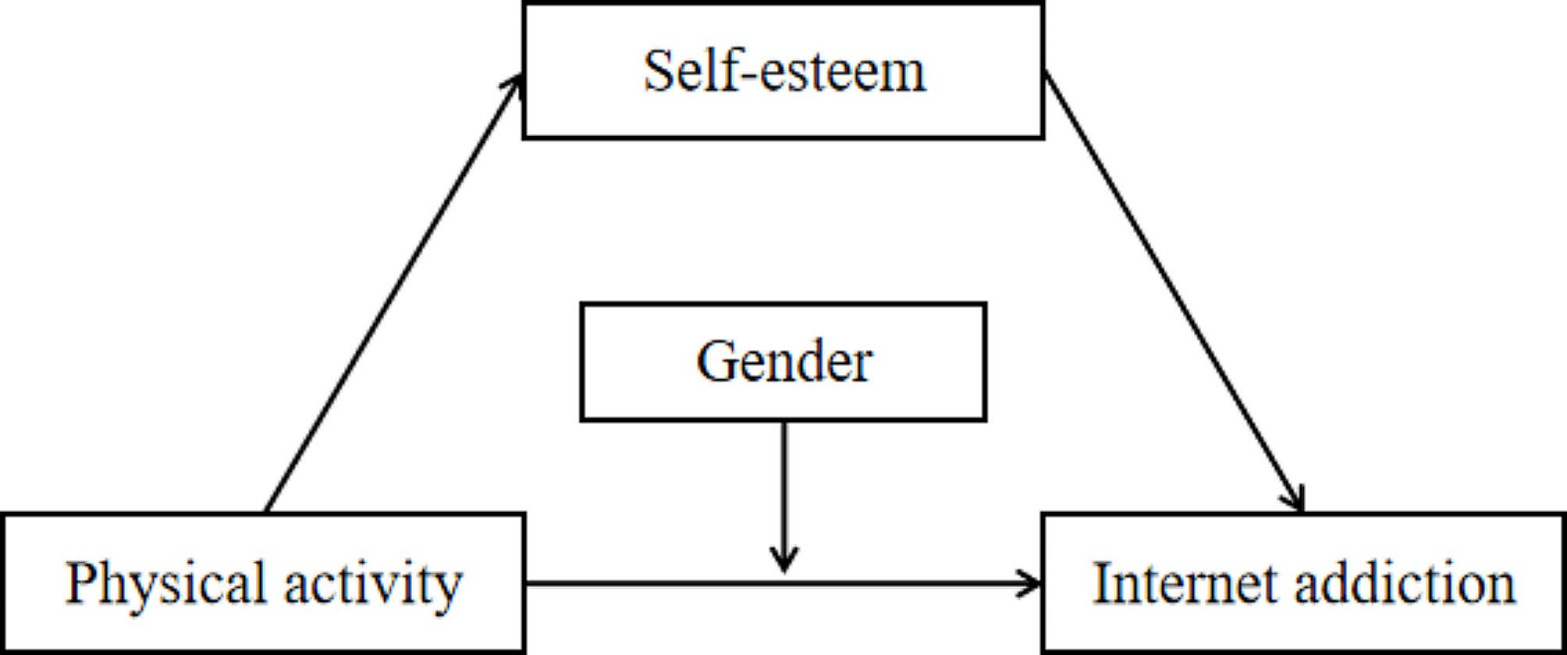
The hypothetical model of the effects of physical activity on internet addiction, with self-Esteem as the mediating variable

Previous empirical studies had demonstrated the predictive effect of physical activity on Internet addiction, but current studies lacked in-depth investigation of the intrinsic mechanism between the two. Therefore, the present study was designed to explore the correlation between self-esteem, physical activity, and Internet addiction in a group of college students, to verify the possible mechanisms of self-esteem between physical activity and Internet addiction, and to examine the gender-multigroup differences in Internet addiction (see Fig. 1 for the conceptual model), with the aim of providing references and guidance for reducing Internet addiction among college students.

## Data sources and methods

### Research subjects

The sampling period for this study was from June 1st to 10th, 2022. Due to the impact of the COVID-19 pandemic in most provinces of China during that time, a convenience sampling method was adopted to select three provinces that were not affected by the pandemic. Simple random sampling was then used to select five universities: Zhengzhou University, Beijing Normal University, Inner Mongolia University of Science and Technology, Henan University of Engineering, and China University of Mining and Technology (Beijing). This study employed a cluster random sampling method, with classes as the unit of sampling, to select 950 college students [19.96 ± 2.69 years old, 497 male students (20.02 ± 3.17 years old) and 358 female students (19.66 ± 1.79 years old ) ] as the research subjects. All participants were between 18 and 24 years old.

### Investigation procedures

**Fig. 2:**
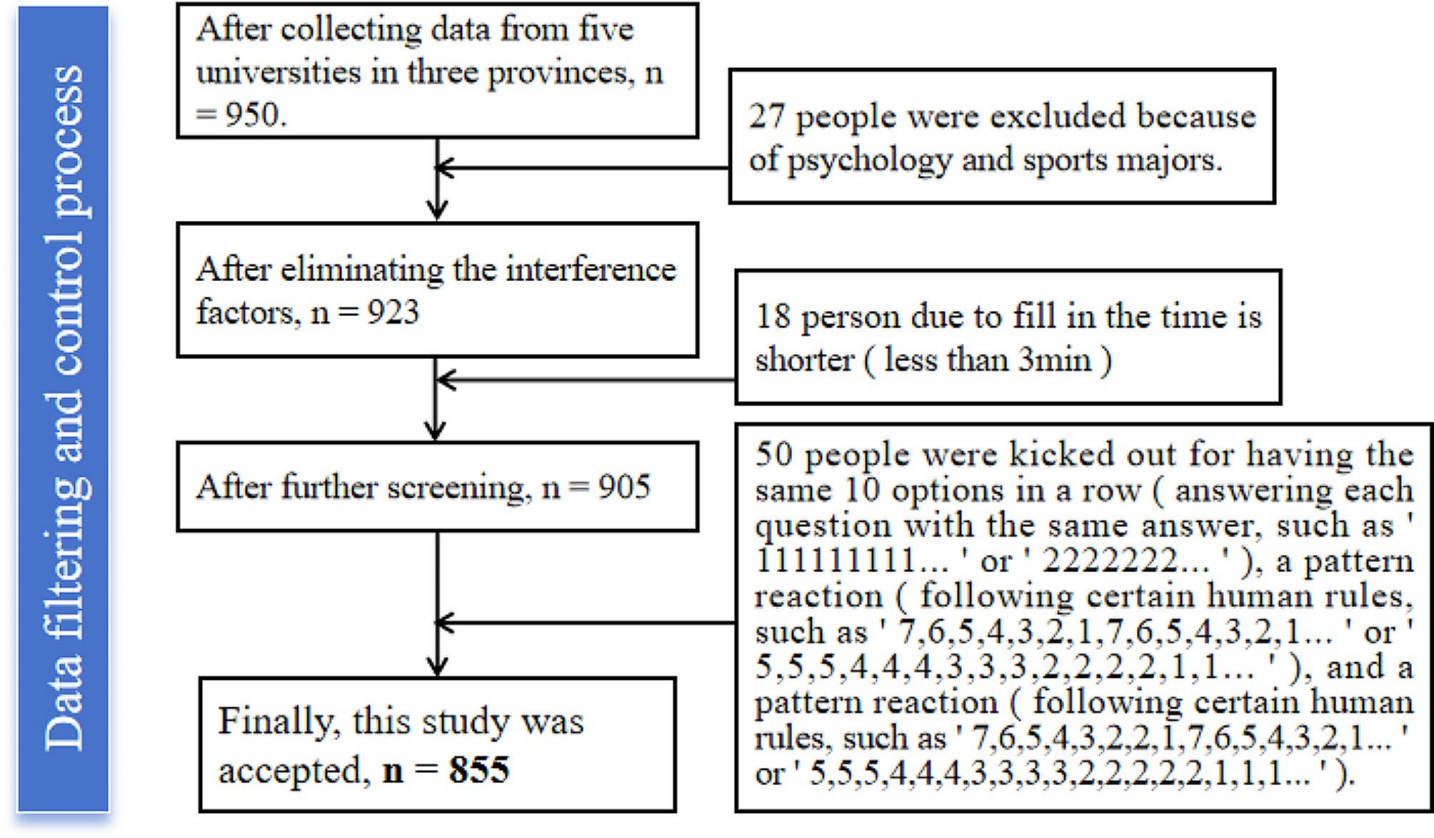
The screening process

In this study, the test was implemented collectively as a class, with two experienced postgraduates serving as the primary examiners for each class. Prior to the implementation of the test, special training (For instance, how to connect with the respective universities, follow the established steps, maintain proper etiquette and courtesy, introduce ourselves when conducting the survey, outline the contents of the questionnaire, explain each item, provide instructions for filling out the questionnaire, time the process, conduct a final check of the completed questionnaires, and ensure confidentiality throughout the entire process.) was given to all examiners. Adolescents participated voluntarily after obtaining the consent of school leaders and teachers in charge. Measurements were conducted anonymously in the classroom, and before carrying out the survey, the test subjects were asked to fill out the questionnaires truthfully followed by instructions according to their real circumstances. All questionnaires were collected on the spot, and the investigator answered any questions asked by the subjects. In the process of administering the survey, the counselor was shunned and the questionnaires were filled out anonymously, without involving personal privacy such as student number. The document was also informed to clearly state that this survey is anonymous, the results of the survey are only used for scientific research, and will not pose any risk to the daily life of the participants, and that the participants’ participation in the study is completely voluntary. Participants were asked to confirm that they had read the document and agreed to participate in the study before continuing the survey. The test was completed in one sitting and took approximately 10 min, and all questionnaires were returned on the spot, with a total of 950 questionnaires distributed. After screening, 855 were valid, resulting in a questionnaire validity rate of 90%. The data screening process is shown in Fig. 2.This study was conducted in compliance with local laws and regulations and institutional requirements. For another, the study was approved by the Ethics Committee of Zhengzhou University (Approval No. ZZUIRB2022-JCYXY0029), with informed consent from all participants. Still, ensuring the voluntary participation and informed consent of all participants during the investigation should be seriously performed.

## Measurement of variables

### Physical activity rating scale (PARS-3)

The scale was developed by the Japanese scholar, Mr. K.Y. Hashimoto [31] and revised by Mr. Liang Deqing et al. [32]. The scale has three dimensions, namely, activity intensity, activity time and activity frequency, with one item for each dimension and a 5-point Likert scale, where the higher the score, the greater the physical activity. The scale is scored by the formula “Intensity × (Time-1) × Frequency = Total Physical Activity Score”, with scores less than or Eq. 19 corresponding to a low level of physical activity, scores ranging from 20 to 42 indicating a moderate level of physical activity, and scores 43 representing a high level of physical activity. The reliability of this scale is high, with a re-test reliability of 0.82, and the total clombach α is 0.774.

### Rosenberg self-esteem scale (SES)

The scale was compiled by Rosenberg in 1965 [14], introduced and translated by Ji Yifu and Yu Xin [33], and widely used in China. There are 10 items, with 1, 2, 4, 6, 7, and 8 as positively scored questions, and the rest as negatively scored questions. Adopting a 5-point Likert scale(By adding a neutral option between the options of a four-point Likert scale, the original four-point Likert scale is transformed into a five-point Likert scale.), the scale has a score range of 10–40, with higher scores indicating higher levels of self-esteem in individuals. The scale mainly measures overall self-esteem, which is simple to understand, easy to score, and is one of the most widely used self-esteem scales. The scale demonstrates good validity, with a retest reliability of 0.72. The total Cronbacha’s alpha of the scale is 0.89.

### Chinese internet addiction scale (revised chen internet addiction scale, CIAS-R)

The scale was developed by Shuhui Chen et al. (2003) [34], with a total of twenty-six questions in five dimensions, namely: COMPULSIVENESS (α = 0.886), WITHDRAWAL SYMPTOMS (α = 0.93), TOLERANCE (α = 0.911), INTERPERSONAL HEALTH PROBLEMS (α = 0.895), and TIME MANAGEMENT PROBLEMS ( α = 0.908). The scale uses a 5-point Likert scale(By adding a neutral option between the options of a four-point Likert scale, the original four-point Likert scale is transformed into a five-point Likert scale.) ranging from “very non-compliant” to “very compliant,” with a higher total score indicating a more severe degree of Internet addiction; If the total score is ≥ 58, the individual is preliminarily screened as a potential Internet addict; if the total score is score is ≥ 68, the individual is diagnosed as an Internet addict. The scale has high reliability and validity, with a retest reliability of 0.83 and a Cronbach’s coefficient of 0.976 for the total scale.

### Statistical methods

In this study, three indicateors of self-esteem, physical activity and Internet addiction were subjected to normality test using SPSS 21.0, results showed that p-values were greater than 0.05, indicating a normal distribution. Then, descriptive statistics, reliability test and Pearson correlation analysis [35] were performed. After the research variables were standardized, mediation effect analysis was performed through the standardized hierarchical multiple regression equations, taking the amount of physical activity as an independent variable, the self-esteem as the mediator variable, and Internet addiction as the dependent variable, and the incremental changes in R^2^ and F values in the results were used to assess the main effects of the study variables. Finally, after standardizing each research variable, the significance of the mediation effect test was evaluated by bias-corrected nonparametric percentile Bootstrap method (5000 repetitive samples) using the macro program process in SPSS [36], and the absence of a confidence interval of 0 indicates significance (*P* < 0.05) [37]. Finally, the equation model conducted moderated mediation effect analysis using physical activity as the independent variable, gender as the moderator variable, self-esteem as the mediator variable, and Internet addiction as the dependent variable.

## Result

### Common method bias test

Data collection in this study was completed by self-assessment, which may introduce common methodological bias. Harman’s one-way test was used to factor analyze all items involved in this study. The results showed that a total of 11 factors with eigenvalues greater than 1 were extracted from the exploratory factor analysis, and the amount of variance explained by the first factor was 26.7%, much lower than the critical value of 40%. This indicateed that the data of this study were not affected by common method bias.

### Gender differences in physical activity and internet addiction

By taking gender as a grouping variable, the amount of physical activity, intensity of physical activity, frequency of physical activity, duration of physical activity, Internet addiction, compulsivity, withdrawal symptoms, tolerance, interpersonal health problems, and time management problems as test variables, the results of the independent samples t-test are shown in Table 1. In terms of physical activity, physical activity intensity, and physical activity frequency, the scores of men were significantly greater than those of women (all *p* < 0.01). In terms of physical activity time, the difference between genders was not significant (*P* > 0.05). In terms of Internet addiction, withdrawal symptoms, interpersonal health problems, and time management problems, the scores of female students were all significantly greater than those of male students (*P* < 0.01). Nonetheless, none of the differences between genders were significant (*P* > 0.05) in terms of compulsion and tolerance.

**Table 1:** Analysis of gender differences in physical activity and internet addiction

Variant		Population (*n* = 855)	Male (*n* = 497)	Female (*n* = 358)	F	T	95% confidence interval	
							Lower limit	Limi-t
Physical Activity		3.82 ± 0.80	3.92 ± 0.80	3.67 ± 0.79	20.90**	−3.01**	0.14	0.36
	1. Intensity of physical activity	3.84 ± 1.00	3.98 ± 0.96	3.64 ± 1.01	24.24**	−4.11**	−0.20	0.47
	2. Frequency of physical activity	3.85 ± 1.01	3.99 ± 1.03	3.66 ± 0.97	21.85**	4.57**	−0.19	−0.46
	3. Physical activity time	3.85 ± 0.89	3.89 ± 0.93	3.60 ± 0.84	2.39	4.92	−0.02	0.22
Internet addiction		3.58 ± 0.62	3.51 ± 0.66	3.67 ± 0.56	15.51**	4.67**	−0.25	−0.87
	1. Compulsive	3.49 ± 0.79	3.46 ± 0.83	3.52 ± 0.73	1.10	1.54	−0.17	0.50
	2. Withdrawal symptoms	3.59 ± 0.64	3.52 ± 0.68	3.70 ± 0.58	16.89**	−4.11**	−0.27	−0.10
	3. Tolerance	3.36 ± 0.98	3.40 ± 1.00	3.31 ± 0.87	1.69	−1.30	−0.45	0.22
	4. Interpersonal health issues	3.57 ± 0.67	3.51 ± 0.71	3.65 ± 0.60	9.03**	−3.01**	−0.23	−0.05
	5.Time management issues	3.66 ± 0.71	3.58 ± 0.74	3.78 ± 0.65	16.93**	−4.12**	−0.30	−0.11

Note **P<0.01

### Analysis of the relationship between physical activity, self-esteem and internet addiction among college students

Using physical activity, self-esteem, and Internet addiction as variables, and age and grade as control variables, a biased correlation bivariate two-sided test shows (Table 2) that physical activity is significantly and positively correlated with self-esteem (*r* = 0.26, *P* < 0.01), and significantly and negatively correlated with Internet addiction (*r*=-0.23, *P* < 0.01), while self-esteem was significantly and negatively correlated with Internet addiction (*r*=- 0.22, *P* < 0.01). The significant correlation between the main variables suggests that mediation effects can be further inspected [27, 38].

**Table 2:** Results of descriptive statistical analysis and correlation analysis of variables (*N* = 855)

Variant	M ± SD	Physical activity	Self-confidence	Internet addiction
Physical activity	3.82 ± 0.80	1.00		
Self-esteem	2.88 ± 0.67	0.26**	1.00	
Internet addiction	3.58 ± 0.62	−0.23**	−0.22**	1.00

Note *p < 0.05, **p < 0.01

### A test of the mediating role of physical activity and internet addiction

Using physical activity as the independent variable X, Internet addiction as the dependent variable Y, self-esteem as the mediator variable M, and other factors as covariates, mediation effect regression analysis was conducted through Model 4 in the Process macro program. The test results in Table 3 showed that the negative predictive effect of physical activity on Internet addiction among college students was still significant (B = -0.14, with a 95% confidence interval of [-0.20, − 0.09], *P* < 0.01). In addition, the amount of physical activity can significantly and positively predict the degree of self-esteem (B = 0.22, 95% confidence interval of [0.17, 0.26], *P* < 0.01), and the degree of self-esteem can significantly and negatively predict the extent of Internet addiction (B = -0.15, 95% confidence interval of [-0.21, -0.09], *P* < 0.01). According to the mediating effect test criterion proposed by Wen Zhonglin [35] as well as the results of the study, it can be indicated that self-esteem is a mediating variable of physical activity which has effect on college students’ Internet addiction, playing a mediating role between the two.

**Table 3:** Regression analysis of the mediating role of self-esteem (*N* = 855)

Implicit variable		Overall fit index			Significance of regression coefficients				
Outcome variable	Predictor variable	R	R^2^	F	β	SE	LLCI	ULCI	t
Self-esteem	Gender	0.32	0.10	23.89	−0.20	0.47	−0.29	−0.11	−4.31^**^
	Age				0.02	0.03	−0.002	0.32	1.69
	Grade				−0.002	0.02	−0.04	−0.04	0.03^**^
	Physical activity				0.22	0.03	0.17	0.26	10.06^**^
Internet addiction	Gender	0.31	0.10	18.07	0.09	0.04	0.01	0.18	2.20^**^
	Age				0.001	0.01	−0.01	0.02	0.14^**^
	Grade				−0.01	0.02	−0.05	0.02	−0.74
	Physical activity				−0.14	0.03	−0.20	−0.09	−5.44^**^
	Self-confidence				−0.15	0.03	−0.21	−0.09	−4.93^**^

Note **p < 0.01

**Fig. 3:**
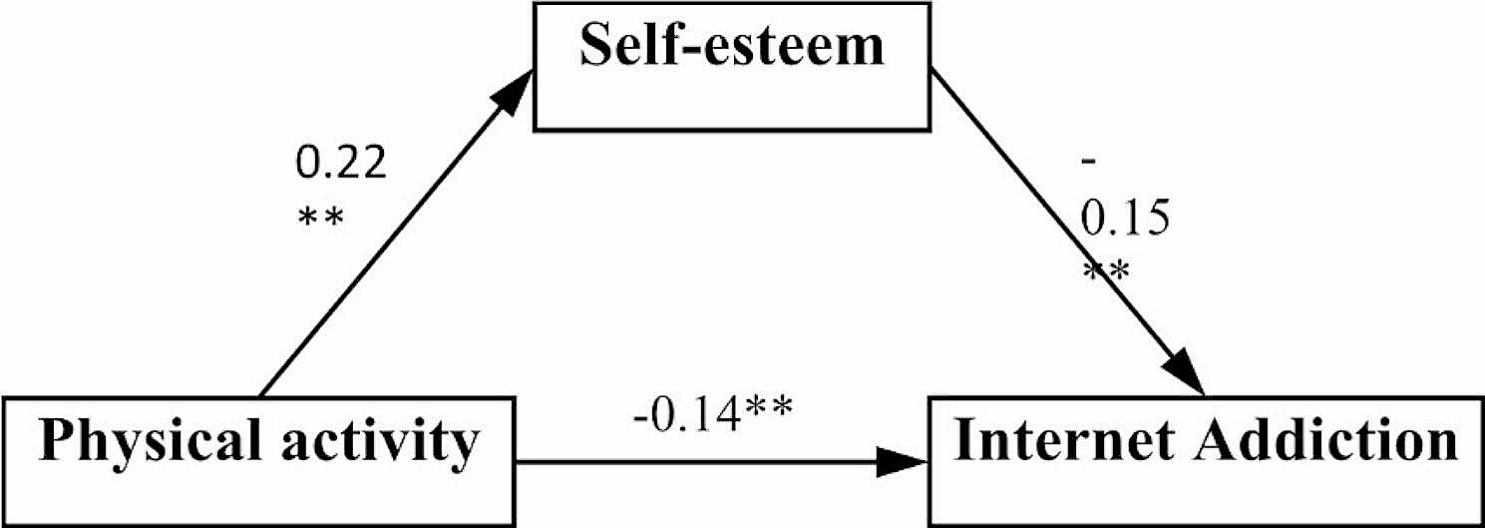
Model of self-esteem as a mediator between physical activity and internet addiction

As can be seen from Table 4, the direct path effect value of “physical activity → Internet addiction” is −0.04 and its 95% confidence interval is [−0.06, −0.02], which does not contain 0, indicating that the path effect is significant. Thus physical activity can directly affect college students’ Internet addiction, while the indirect path effect value of “physical activity → self-esteem → Internet addiction” is −0.15, and its 95% confidence interval is [−0.20, −0.10] which excludes 0, indicating that the path is also significant. The total indirect effect value is −0.19, and its 95% confidence interval [−0.21, −0.10] does not include 0, indicating that this path is also significant. Given the above, physical activity can not only directly reduce college students’ Internet addiction, but also indirectly affect their Internet addiction by moderating their self-esteem, where self-esteem plays a mediating role between physical activity and college students’ Internet addiction, with the mediating effect accounting for 78.95% of the totality, as shown in Fig. 3.

**Table 4:** Bootstrap analysis of mediation effect significance test

Type of Effect	Path	Effect Size	BootSE	Boot95%CI		Effect Size Ratio
				Lower Limit	Upper Limit	
Indirect effect	Physical activity → self-esteem → internet addiction	−0.15	0.026	−0.20	−0.10	78.95%
Direct effect	Physical activity → Internet addiction	−0.04	0.01	−0.06	−0.02	21.05%
Total Effect	(Physical activity → self-esteem → internet addiction)+(Physical activity → Internet addiction)	−0.19	0.026	−0.21	−0.10	100%

### The regulatory role of gender

With physical activity as the independent variable X, Internet addiction as the dependent variable Y, self-esteem mediator variable M, gender as the moderator variable Z, and other factors as covariates, model 5 in the Process macro program was used to conduct the moderated mediating effect, and the results showed that the product of physical activity and gender significantly predicted Internet addiction (β = 0.181, t = 2.99, *P* < 0.01), suggesting that gender plays a moderating role between physical activity and internet addiction, as shown in Table 5.

**Table 5:** Regression analysis of gender regulation

Dependent variable		Overall fit index			Significance of regression coefficients				
Outcome variable	Predictor variable	R	R^2^	F	β	SE	LLCI	ULCI	t
Internet addiction		0.324	0.105	90.63^**^					
	Physical activity				−0.14	0.03	−0.19	−0.09	−5.41^**^
	Self-esteem				−0.41	0.09	−0.58	−0.23	−4.51^**^
	Gender				−0.42	−0.18	−0.76	−0.07	−2.38^*^
	Product of physical activity and gender				0.18	0.06	0.06	0.30	2.99^**^

**Fig. 4:**
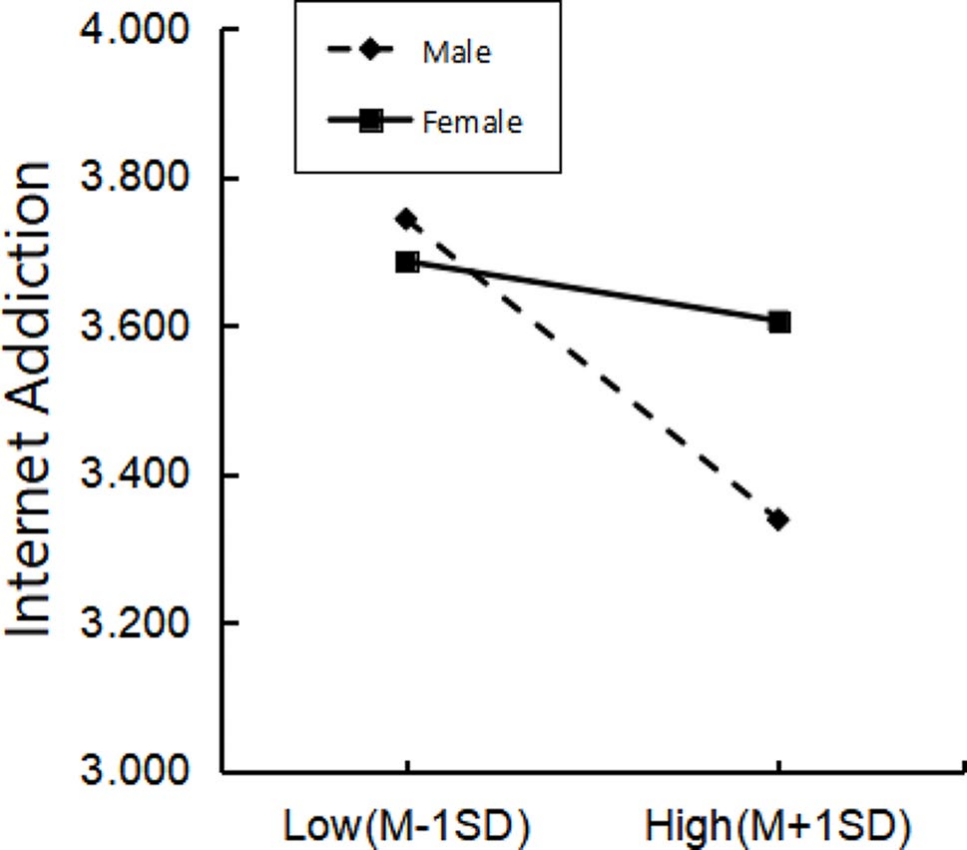
Moderating effect of gender on the relationship between physical activity and internet addiction

To further explore the moderating role of gender in the relationship between physical activity and internet addiction among college students, a simple slope analysis was conducted. The results indicate that gender significantly moderates the relationship between physical activity and internet addiction, suggesting that the relationship pattern differs between male and female groups. When the level of physical activity is low, male students tend to have slightly higher levels of internet addiction than females. However, as the level of physical activity increases, the internet addiction of male students (simple slope = −0.17, t = −5.08, *P* < 0.001) decreases at a faster rate compared to female students (simple slope = −0.10, t = −2.6, *P* < 0.05), indicating that male students’ internet addiction significantly decreases in comparison to females when the level of physical activity is higher. The results are shown in Fig. 4.

## Discussions

Current international research has not found any study that directly explores the mediating role of self-esteem in the relationship between physical activity and Internet addiction, all of which imply, either directly or indirectly, that physical activity may increase individual’s self-esteem, and that the level of self-esteem is negatively correlated with Internet addiction. Previous studies have explored the role of self-esteem in Internet addiction from other psychological perspectives. This study examines the differences in physical activity and Internet addiction between genders among Chinese college students. Through multiple linear regression analysis, the study verifies the correlation between physical activity, self-esteem, and Internet addiction among college students. Furthermore, the study investigates the mediating role of self-esteem between physical activity and Internet addiction, and explores whether gender plays a moderating role in the model.

### Gender differences in physical activity

The present study shows that male college students generally score higher than female students in physical activity, which is consistent with the findings of Aubert et al. [39]. This result further validates the previous findings, all of which concluded that males are more inclined to participate in physical activities compared to females. Social role theory suggests that people play different roles in different social situations [40]. Traditionally, men are expected to play more energetic, adventurous, and competitive roles, while women are expected to stay quiet, refined, and soft. This role expectation may make men more inclined to participate in physical activities in order to fulfill the social expectations placed on them.

### Gender differences in internet addiction

The present study finds that the scores of female students are significantly greater than those of male students in terms of Internet addiction, which is contrary to the study of Chen et al. [41], whose study also compared the differences in Internet addiction between genders using Chinese college students as the subject of the study, and all results showed that male students had significantly higher scores of Internet addiction than females. However, the present study is in line with the findings of Aylaz et al. (2016), which revealed that female high school students in Turkey had significantly higher Internet addiction scores than male students [42]. Additionally, Tang et al. (2014) showed that the prevalence of social network addiction among males and females was 27.8% and 37.3% respectively, with a significantly higher prevalence among females [43]. Reasons for this situation may be that, on the one hand, according to the statistical report on China’s Internet development, female Internet users accounted for 39.6% of the total number of Internet users in the country in 2004, indicating that the number of female Internet users was significantly lower than that of males in the early stage of the development of the Internet. However, by 2020, female Internet users accounted for 49% of total Internet users, and the proportion of male and female Internet users tended to be the same, which indicates that in the past 15 years, the number of female Internet users has been gradually increasing, with females engaging in online activities more frequently. This may be an important reason for the yearly rise of female Internet addiction symptoms. On the other hand, some studies have found that gender is a key factor explaining why individuals become addicted to the Internet in different ways [44]. Females have a greater preference for online interpersonal interactions and online shopping consumption than males [45]. In the past 15 years, China has seen a rise in online shopping consumption and social media platforms. Chinese women spend a lot of time interacting with friends and family on newly emerging social applications such as WeChat or TikTok [46], and also make online purchases through e-commerce applications such as Amazon and Alibaba [46]. These various online behaviors may lead to the increasing likelihood of Internet addiction among female college students. Additionally, this could be closely related to the personality traits of male students. Research has found that compared to females, males tend to be more open-minded and optimistic, while females are more prone to experiencing depression [47, 48]. According to the theory of needs satisfaction, when adolescents’ psychological needs are not met in their real lives, they are more likely to seek happiness, comfort, and fulfillment in the virtual world of the internet, leading to a vicious cycle of addiction [49]. Therefore, when male students have higher levels of physical activity, and they are aware of their interests and hobbies, they are more likely to resist the temptations of online gaming and other aspects of the internet, resulting in a lower risk of problematic internet behavior compared to females, which is consistent with previous research.

### Physical activity negatively predicts internet addiction

The results of this study showed that physical activity negatively predicts Internet addiction. This aligns with a meta-analysis conducted by Wu Jin [12, 13], which includes a large number of RCTs of exercise intervention for Internet addiction. The final results indicate that physical activity intervention can effectively reduce the level of Internet addiction in adolescents, which not only improves the physical condition of addicts, but also helps to enhance self-esteem, hone the will, and resistance to Internet addiction. Both the Internet and physical activity provide participants with a sense of interaction and entertainment. The difference lies in the fact that the participatory use of the Internet immerses individuals in the virtual world it brings, whereas physical activity allows participants to experience the real world by guiding and building their physical and mental well-being. One of the theoretical underpinnings of exercise interventions is that engaging in physical activity occupies the time spent on the Internet, while at the same time subconsciously brings positive physiological and psychological changes to the participant. Therefore, adolescents who are physically active on a regular basis usually have good physical and mental health, and healthier Internet use behaviors [50].

### Self-esteem mediates the role of physical activity in college students’ internet addiction

In this study, self-esteem is significantly positively correlated with physical activity and significantly negatively correlated with Internet addiction. Furthermore, self-esteem partially mediates the relationship between physical activity and Internet addiction among college students, accounting for 78.95% of the total effect. This suggests that physical activity can reduce college students’ Internet addiction index by improving individual self-esteem, in which self-esteem exists as a mediating factor. The positive effects of physical activity on the body and mind have been confirmed [51], and individuals who often engage in physical activity have higher level of self-esteem and positive psychological emotions and states [52], while those with low self-esteem are often prone to fall into doubt about their own abilities and may turn to the Internet as a way of hiding from the reality and seeking psychological compensation [53]. Physical activity has the effect of shaping the human body, improving mental image, and promoting the health of the individual, which may have a positive effect on individual’s well-being, and thus enhance self-esteem. This finding is also consistent with the results of Sun Chao’s study [17]. For another, in Kumer’s study, a survey on self-esteem and Internet addiction among 200 college students, found that those with low self-esteem have a higher degree of Internet addiction, and those with high self-esteem have a lower tendency to Internet addiction, indicating a significant negative correlation between self-esteem and Internet addiction [20]. Therefore, after self-esteem is raised, individuals may have more time and energy to present themselves and participate more in activities such as socializing, which further reduces the symptoms of Internet addiction. If individuals are less involved in physical activities, their health status and physical shape may be unsatisfactory, affecting the level of their self-esteem. Therefore, they may reduce their social interaction and find self-satisfaction in the virtual network, leading to the increase of Internet addiction.

## The regulatory role of gender

The present study finds the moderating role of gender in the relationship between physical activity and Internet addiction. At the same time, the moderating role of gender should also be paid attention to when preventing and intervening in the Internet addiction of college students, and thus different strategies and methods for male and female college students should be adopted. For male college students, they should participate in more physical activities, divert their attention gradually increase the frequency, time and intensity of physical activities, and enhance the sense of gain in sports in order to reduce the use of cell phones. While for female college students, they should strengthen the social interaction in reality, change the form of participation in sports, and participate in group-oriented, collaborative, and different contextual sports programs, so as to enhance the attentional engagement in sports, reduce the dependence on the Internet and better guide their rational use of the Internet. As a result, guiding students to use the Internet reasonably is crucial for their achievement of emotional release in physical activity.

The above findings support and extend the theory of physical activity treatment for Internet addiction, provide evidence for exercise intervention programs on Internet addiction. Meanwhile, the findings have theoretical and practical implications for the treatment of Internet addiction in college students, especially for the idea of suppressing addictive behavioral impulses according to the influence of pathway from physical activity to Internet addiction. The individual factors of Internet addiction, such as behavioral constraints, can be managed by cultivating regular physical behavioral habits, thus enhancing individual self-esteem, effectively preventing the addictive impulsivity, and abstaining from addictive online behaviors.

### Disadvantages

First, in this study, the measures of physical activity, self-esteem, and Internet addiction were taken from the self-assessment reports of college students, which may result in a slight deviation from the actual situation. However, the study tried to ensure standardized testing procedures, and removed as many invalid questionnaires as possible after the tests were completed, minimizing the bias and maintaining the reliability of the findings. Second, sampling bias may exist in the study. The random distribution of the questionnaire resulted in a biased sample in terms of gender, with more males and fewer females, as well as uneven differences in place of origin, grade, age, etc. Consequently, a sampling bias that may have had some impact on the external validity of the results. Thirdly, this study only used cross-sectional research without longitudinal tracking to explore the changing mechanism of influencing factors on Internet addiction among college students, which may be considered as a future research direction. Additionally, a small R2 value may be due to insufficient sample size or other factors. However, the model’s p-value is less than 0.01, indicating that the model is highly significant. Subsequent research will aim to expand the sample size for further investigation.

## Conclusion

In terms of physical activity, the scores of male students are significantly higer than those of females, while in terms of Internet addiction, female students gain significantly higher scores than males. The findings prove that physical activity not only directly improves the symptoms of Internet addiction with gender playing a moderating role in this pathway, but also indirectly reduces the Internet addiction among college students by enhancing individual self-esteem.

## Data Availability

The data for this study are openly available and transparent, if there is a need for their use, please contact the author via email at dzh_cumt_edu@163.com.

## Declarations

### Publication statement

All claims expressed herein are those of the authors only and do not necessarily represent those of their affiliated organizations, the publisher, editors, and reviewers. Any products that may be evaluated herein, or statements that may be made by their manufacturers, do not have any guarantees or endorsements the publisher.
